# A Simplified GIS Approach to Modeling Global Leaf Water Isoscapes

**DOI:** 10.1371/journal.pone.0002447

**Published:** 2008-06-18

**Authors:** Jason B. West, Adam Sobek, James R. Ehleringer

**Affiliations:** 1 Department of Biology, University of Utah, Salt Lake City, Utah, United States of America; 2 Department of Geography, University of Utah, Salt Lake City, Utah, United States of America; Centre National de la Recherche Scientifique, France

## Abstract

The stable hydrogen (δ^2^H) and oxygen (δ^18^O) isotope ratios of organic and inorganic materials record biological and physical processes through the effects of substrate isotopic composition and fractionations that occur as reactions proceed. At large scales, these processes can exhibit spatial predictability because of the effects of coherent climatic patterns over the Earth's surface. Attempts to model spatial variation in the stable isotope ratios of water have been made for decades. Leaf water has a particular importance for some applications, including plant organic materials that record spatial and temporal climate variability and that may be a source of food for migrating animals. It is also an important source of the variability in the isotopic composition of atmospheric gases. Although efforts to model global-scale leaf water isotope ratio spatial variation have been made (especially of δ^18^O), significant uncertainty remains in models and their execution across spatial domains. We introduce here a Geographic Information System (GIS) approach to the generation of global, spatially-explicit isotope landscapes ( = isoscapes) of “climate normal” leaf water isotope ratios. We evaluate the approach and the resulting products by comparison with simulation model outputs and point measurements, where obtainable, over the Earth's surface. The isoscapes were generated using biophysical models of isotope fractionation and spatially continuous precipitation isotope and climate layers as input model drivers. Leaf water δ^18^O isoscapes produced here generally agreed with latitudinal averages from GCM/biophysical model products, as well as mean values from point measurements. These results show global-scale spatial coherence in leaf water isotope ratios, similar to that observed for precipitation and validate the GIS approach to modeling leaf water isotopes. These results demonstrate that relatively simple models of leaf water enrichment combined with spatially continuous precipitation isotope ratio and climate data layers yield accurate global leaf water estimates applicable to important questions in ecology and atmospheric science.

## Introduction

Many ecological questions are concerned with detecting and quantifying the movement of materials or organisms across space and time. The components tracked can be inorganic or organic compounds, individual organisms, or populations or communities of organisms moving between locations in soils, within and among forest canopies, along elevation gradients or across landscapes. Considering stable isotope ratio variation in a spatial context has allowed the quantification of many aspects of these movements when other tools were not able to provide this information [1], [2], [3].

Plants record aspects of their environment in the stable isotope ratios of their tissues and can provide geographic and climatic information [4]. This information is useful for a range of ecological questions since plants are sources of animal food, plant species movements across landscapes may occur as a result of a variety of factors, including climate change, and plant water use and leaf water isotopic enrichment significantly affect the isotopic composition and dynamics of the atmosphere. A potential wealth of information is available in the spatio-temporal variation of plant stable isotopes. We present here a Geographic Information System (GIS) approach to the production of spatially continuous stable isotope landscapes (hereafter “isoscapes”) of global leaf water δ^18^O and δ^2^H for use in a wide range of ecological and atmospheric research. These isoscapes are based on biophysical models of leaf water isotopic enrichment that are executed in a GIS modeling framework. Spatially explicit model predictions of the isotopic composition of leaf water and other biosphere and atmosphere pools have been made for some time using various platforms and approaches [5], [6], [7]. We present the GIS approach as novel and complimentary to other modeling efforts designed to make similar predictions. We believe that this approach provides a streamlined platform for modeling, sharing and integrating spatial data, and as such provides a unique entry point to the rich potential in modeling and interpreting spatial variation in stable isotope ratios [e.g.8], [9], [10], [11], increasing the potential for collaborative and innovative research through the development and application of isoscapes. In addition to introducing the approach we present model comparisons (illustrating their use as a model diagnostic tool) and a comparison with existing point measurements of leaf water data in order to evaluate the accuracy of the modeled isoscapes.

The isoscapes are produced using models that mechanistically describe the evaporative enrichment of leaf water δ^18^O and δ^2^H during transpiration [e.g.12], [13], [14], [15], [16], [17], [18], [19]. Although these models find strong support from observations at the leaf level [13], [14], [16], there remain large uncertainties, including the accuracy of the models themselves and the paucity of data needed to drive and test the models at larger scales (e.g., the isotope ratios of atmospheric vapor). Global general circulation models (GCM) that require leaf water δ^18^O e.g.[6], [20] have employed analogous leaf-level models, but unfortunately their estimates of leaf water isotopic enrichment show considerable disagreement [6], [7], [21]. Given the strong interest in spatial variability in plant oxygen and hydrogen isotope ratios because of their application to a wide range of questions [4], we use these systems as a test case to evaluate the utility of this new approach and for providing a platform for comparing distributed data to spatially explicit models.

## Methods

### Leaf water δ^18^O and δ^2^H models

We implemented three steady-state models of leaf water δ^18^O and δ^2^H: one modeling the sites of evaporative enrichment inside leaves (based on the formulations of Craig & Gordon [22]; see http://isoscapes.org for detailed GIS and modeling descriptions) and two models of “bulk” leaf water δ^18^O and δ^2^H that take into account isotopic heterogeneity within leaves. It is important to note that non-steady state models of leaf water enrichment have been described [23] and have important explanatory power in some cases [c.f.24], [25]. However, explicit non-steady-state dynamics were not modeled here. Given our currently limited data and understanding of the importance of non-steady state dynamics for large scale questions (e.g., how biomes differ in non-steady-state dynamics), as well as very limited capacity to parameterize these models for large landscapes, we utilized steady-state models here, recognizing that important variability (e.g., diurnal changes in leaf water isotopic composition) is not captured. It should also be noted that Cuntz et al. [5] have modeled non-steady-state dynamics at the global scale by incorporating a lag component to leaf responses to changing climate into their model of the δ^18^O of atmospheric CO_2_.

### Leaf water δ^18^O and δ^2^H at sites of enrichment

It is first assumed that there is no fractionation with water uptake from the soil [14], [26], and that negligible fractionation occurs as the water moves through the plant to the evaporating surfaces inside the leaves (xylem water = soil available water). The water in the leaf then experiences isotopic enrichment based on phase change ( = equilibrium) and diffusion ( = kinetic) processes. Equilibrium fractionation (α^*^) is temperature dependent and is described as follows:
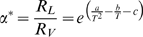
(1)where *e* is Euler's number (not vapor pressure as below with subscripts), *R_L_* is the liquid water isotope ratio (^2^H/^1^H or ^18^O/^16^O), *R_V_* is the water vapor isotope ratio, and *T* is temperature in degrees Kelvin. For oxygen *a* = 1137, *b* = 0.4156, *c* = 0.0020667 and for hydrogen *a* = 24844, *b* = 76.248, and *c* = 0.052612 [27]. Kinetic fractionation is described for diffusion from the evaporating surface inside the leaf to the atmosphere, taking into account diffusion through the leaf boundary layer. The kinetic fractionation factor has been estimated as *α_k_* = 1.032 for oxygen and *α_k_* =  1.0164 for hydrogen [28]; revised from [29], and for diffusion through a boundary layer is *α_kb_* = 1.021 and *α_kb_* = 1.011 for hydrogen [14]. The full equation for steady state leaf water enrichment is:
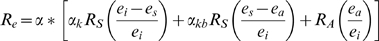
(2)where *R_e_* is the isotope ratio of evaporatively enriched leaf water, *R_S_* is the isotope ratio of the source water, *R_A_* is the isotope ratio of the atmospheric water vapor, *e_i_* is internal leaf vapor pressure, *e_s_* is the leaf surface vapor pressure, and *e_a_* is atmospheric vapor pressure. Leaf surface vapor pressure is estimated using equations developed by Ball [30] from stomatal conductance and transpiration rate. Predicted leaf water isotope ratios (*δ*) are then expressed as parts per thousand or per mil (‰) relative to the isotope standard “Standard Mean Ocean Water” (SMOW):
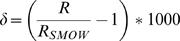
(3)where *R* is the ratio of the heavy to light isotope (^18^O/^17^O or ^2^H/^1^H) in the sample and *R_SMOW_* = 0.0020052 for oxygen and *R_SMOW_* = 0.00015576 for hydrogen [31], [32].

### Bulk leaf water δ^18^O and δ^2^H models

Models of leaf water enrichment that are based on these formulations of Craig & Gordon [22] routinely overestimate measured leaf water δ^18^O [14], [33], [34] though they can also underestimate it [12], [35]. We evaluated two alternative models of “bulk” leaf water that have been developed to explain this discrepancy. The first model is a simple “two-pool” model where bulk leaf water was assumed to be composed of 90% evaporatively enriched water, and 10% un-enriched water [13], [14], [15], [36]. We note that a mathematically identical, but conceptually different model assumes that some fraction of the leaf water has not reached steady-state at the time of measurement [14].

It has been suggested that part of the explanation for the discrepancy between modeled and measured leaf water enrichment is due to the opposing effects of convective flow of un-enriched water to the sites of evaporation, and the simultaneous back diffusion of enriched water during transpiration [the Péclet effect; 7]. The Péclet effect has been explicitly modeled for oxygen isotopes in water [16] and is described by the following dimensionless number:
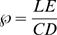
(4)where *L* is the effective path length between the site of evaporation and the un-enriched source water, *E* is the evaporation rate (mol m^−2^ s^−1^), *C* is the molar density of water (55.5×10^3^ mol m^−3^), and *D* is the diffusivity of the H_2_^18^O in water (2.66×10^−9^ m^2^ s^−1^). The path length cannot currently be directly measured, and so in practice is estimated based on the difference between actual bulk leaf water enrichment and that predicted by Δ_e_. Species apparently vary in their effective path length [16], [37]. We use an effective path length of 20 mm, recognizing that ranges as wide as 4 to 166 mm have been reported [37].

Barbour et al. [16] have incorporated the Péclet effect into a modified Craig-Gordon model of leaf water δ^18^O following Farquhar and Lloyd [38] by expressing leaf water as:
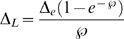
(5)where the Δ subscripts are *L* = bulk leaf water, *e* = evaporatively enriched water (determined from Craig-Gordon formulations). The use of Δ in this case represents enrichment over source water and is expressed as:
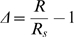
(6)where *R* is ratio of ^18^O to ^16^O in the leaf water and *R_s_* is the ratio of ^18^O to ^16^O in the source water. As with δ, Δ is often expressed as per mil (‰) and multiplied by 1000. In order to make direct comparisons between the two models, Δ_L_ is converted to δ_L_ by combining Equation 6 with Equation 3. It should be noted that water compartmentation, non-steady state effects, and Péclet effects are not mutually exclusive and the exploration of the controls on leaf water enrichment are ongoing [e.g.39], [40]. These models are compared here to allow an exploration of the effects of the assumptions of the models on predicted geographical patterns of leaf water δ^18^O and δ^2^H.

Because of the strong dependence on transpiration rates of the Péclet model, and because unreasonably high transpiration rates would otherwise be predicted for arid zones, stomatal conductance could not be assumed to be constant. Although stomates respond to several stimuli, and these responses remain the subject of some debate [c.f., 41], stomatal conductance (*g_s_*) generally declines with increasing vapor pressure deficit (*D*), apparently in response to changes in leaf water content [42], [43]. Oren et al. [42] have demonstrated that a modification of Lohammar's function [44]:
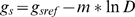
(7)applies generally to a wide range of species and scales of measurement, and that m and g_sref_ (stomatal conductance at *D* = 1 kPa) are well correlated with an average slope of 0.6 [see also 45], [46]. We therefore incorporated Oren et al.'s [42] modification of Lohammar's function into the models described above by substituting 0.6*g_sref_ for *m* in order to allow stomatal conductance to decline as vapor pressure deficit increased across landscapes. A *g_sref_* of 100 mmoles H_2_O m^−2^ s^−1^ was selected for the model runs as reasonable based on the results of Oren et al. [42].

### Model inputs

In order to make global, spatially continuous predictions for leaf water stable isotope ratios, we implemented these mechanistic models of leaf water enrichment in ArcGIS software (ESRI Corporation, Redlands, CA). Essentially the steady-state models are executed repeatedly for each grid cell of input to result in model output that matches the spatial extent and resolution of the input(s). Four general classes of input raster layers were utilized: annual average source water isotope ratios, monthly air temperatures, monthly relative humidities, and elevation (for estimating barometric pressure). Plant source water isotope ratios were estimated with 10 arc-minute (0.1667°) annual average precipitation grids supplied by G. Bowen using the methods described in Bowen & Revenaugh (2003). These precipitation isoscapes should reflect the long-term average isotopic composition of soil water [7]. Actual plant source water isotope ratios can of course vary seasonally and between species due to interactions between ground water, precipitation and runoff, evaporation from the soil surface, differences in rooting depth, and irrigation if transported over long distances [47], [48], [49], [50]. Models of depth-resolved soil water isotopic composition have been developed [51], [52]. However, these intensive modeling efforts include uncertainties in their parameterization, potentially limiting their extensibility to larger regions [51], or assume uniformity of soils globally [52]. Our approach is designed to produce global, long-term average leaf water isotopic composition for comparison with other modeling efforts, especially allowing interaction with other spatially continuous products such as those derived from satellite data. We therefore assume here that global average plant source water is well represented by long-term average precipitation isotope ratios and then test that assumption with comparisons to other model outputs and point measurements of leaf water isotope ratios. We note also that although monthly precipitation grids are available, they have significantly lower data density and therefore inherently larger confidence intervals [53] and so are not utilized here.

For the necessary climate drivers, we employed the “Ten Minute Climatology” monthly grids produced by the Climate Research Unit [CRU; 54], and supplied electronically (http://www.cru.uea.ac.uk/cru/data/tmc.htm) for the temperature and relative humidity inputs. The climate grids are the product of a sophisticated interpolation of global station data from the World Meteorological Organisation normal period of 1961–1990 [54] and should reasonably approximate grid-cell average climate. Since leaf and canopy temperature relevant to calculating leaf water δ^18^O and δ^2^H are not likely well-represented by monthly mean temperature, we estimated grid cell air temperature as mean monthly temperature plus a fraction of the daily temperature range following Hoffmann et al. (2004) where: T_new_ = T_mean_+(0.09 * T_mdr_) and T_new_ is the new air temperature, T_mean_ is the mean monthly air temperature, and T_mdr_ is the monthly mean of the daily air temperature range. The value 0.09 is the median of values fitted by Hoffmann et al. (2004). Leaf temperature is further assumed to be 5% warmer than the air temperature [55]. The calculation of transpiration (for use in the calculation of leaf surface vapor pressure, see Equation 2) also requires an estimate of barometric pressure, so an additional elevation raster of the surface of the Earth (derived from ETOPO-2 and supplied by the National Oceanic & Atmospheric Administration: http://www.ngdc.noaa.gov/mgg/gdas/gd_designagrid.html) was obtained to allow this calculation [56].

### GIS modeling of Isoscapes

The primary assumptions (in addition to those implicit in model structure and coefficient accuracy) were as follows: (1) long-term average plant source water isotope ratios at grid-cell resolution was accurately described by the gridded annual average precipitation isotope ratio map, (2) the climate data sets accurately represented long-term average climatic environments for all twelve months, and (3) vapor δ^18^O and δ^2^H are in isotopic equilibrium with source water δ^18^O and δ^2^H (vapor isotope ratios predicted at unmodified mean air temperature assuming equilibrium with precipitation). These assumptions could be violated for a given set of circumstances or location, and the potential degree of violation is not currently well constrained. Vapor isotopic composition in particular has been observed in disequilibrium from source water on a daily time scale, but may exhibit reasonable equilibrium at longer time scales [57], [58], [59], [60], [61]. Uncertainty about vapor isotopic composition is perhaps most likely to cause significant error in all models, but we emphasize that the model product is long-term average leaf water isotope ratios potentially reducing the overall importance of transient disequilibria. We believe that the assumptions are reasonable for the scale of variation we modeled. Grid cells for which the monthly mean air temperature was less than 0.1°C were eliminated from the monthly predictions. This filter conservatively eliminated spurious leaf water predictions. In order to allow comparison with products designed to understand atmospheric gas isotopic composition that produce productivity-weighted leaf water δ^18^O predictions, the monthly output grids were averaged and weighted by net primary productivity (NPP). The NPP layer employed was obtained from the ISLSCPII project (http://islscp2.sesda.com/ISLSCP2_1/html_pages/islscp2_home.html) and is the average of seventeen global model outputs [62]. Although significant uncertainties remain in the modeling of Earth's NPP, this product represents a “consensus” prediction that at a minimum allows comparison of our estimates with other productivity-weighted model outputs.

## Results

**Figure 1:**
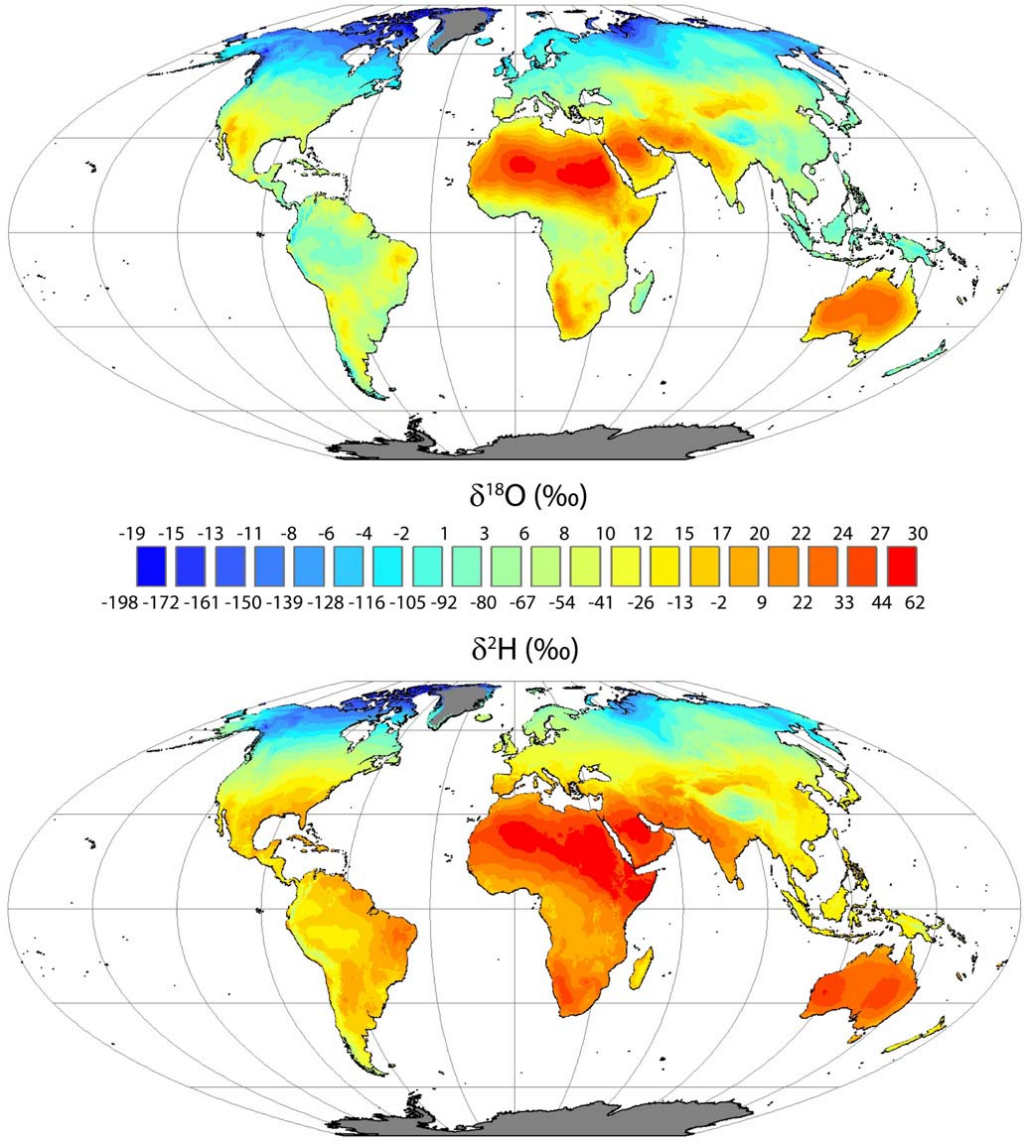
Global mean annual average leaf water δ^18^O and δ^2^H isoscapes for the sites of evaporation within leaves (Flat Polar Quartic projection; Two-pool and Péclet models gave similar, less enriched results). Means were derived from monthly model predictions that utilized input grids of annual average precipitation isotope ratios as plant source water, elevation (for barometric pressure), and modified monthly climate grids for temperature and humidity from the WMO climate normal period (New et al. 2002; see text for details). Grid cells where monthly temperature averages were never above freezing resulted in blank cells (shown as gray).

**Figure 2:**
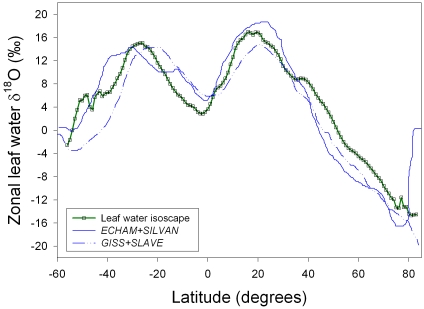
Annual mean leaf water δ^18^O as predicted by the sites of evaporation model (green line: GIS model), and compared to published zonal averages from two GCM+LSM model outputs that also used a Craig-Gordon formulation to predict leaf water δ^18^O (blue lines: *GISS+SLAVE* and *ECHAM+SILVAN*; Hoffmann et al., 2004). The differences between the Hoffmann et al. (2004) models result from both the isotope ratio values generated in the GCMs and the alterations of estimated leaf temperature necessary to fit the modern Dole effect.

The monthly estimates of leaf water δ^2^H and δ^18^O showed spatial and temporal variability consistent with spatial variation in the global, seasonal changes in the climate drivers (individual monthly results not shown, grids available at http://isoscapes.org). The unweighted global leaf water annual average isoscapes at the sites of evaporation are shown in Figure 1 (means of the twelve monthly output grids for both δ^18^O and δ^2^H). The spatial patterns predicted by the Two-pool and Péclet models of bulk leaf water were similar to those shown for the sites of evaporation model (see Figure 2 for latitudinal trends in δ^18^O for all models). Weighting the annual average isoscapes by annual net primary productivity resulted in global average leaf water for sites of evaporation within leaves of δ^18^O = 6.5‰. For bulk leaf predictions that included leaf heterogeneity lower values were predicted: Two-pool = 5.1‰ and Péclet = 4.8‰. Unfortunately leaf water δ^2^H has not been as well studied as δ^18^O, making analogous comparisons for hydrogen not currently possible.

Latitudinal mean δ^18^O values calculated for the site of evaporation are shown in Figure 2 at one-degree intervals. Also plotted are model output results from Hoffmann et al. [6] for the combined general circulation/biochemical models GISS/SLAVE and ECHAM/SILVAN (all model outputs represent un-weighted leaf water isotope ratio means). As discussed by Hoffmann et al. (2004), the GCMs differ in their predictions for precipitation and vapor isotopic composition, and differences in the LSMs result in differences in a fitted parameter that affects leaf temperature. However, generally consistent with these model runs, moving north from the southern edge of the land surface, predicted values from all models climb until approximately 25°S, where they begin to decline until approximately the equator where they begin to increase again until approximately 20°N, where they again decline continuously across the remaining land surface. Although model agreement is greatest at northern latitudes there are significant divergences evident across the latitudinal range. The model outputs produced here resulted in higher leaf water δ^18^O values than the GCM/LSM models north of the tropics. However, the GIS model predictions either fell between the GCM/LSM models or were lower than both for all other latitudes. Not shown in Figure 2 are other model outputs available in the literature [e.g.], [5,63] that, while showing similar latitudinal patterns, also show disagreement with the GCM/LSM and GIS model predictions.

**Figure 3:**
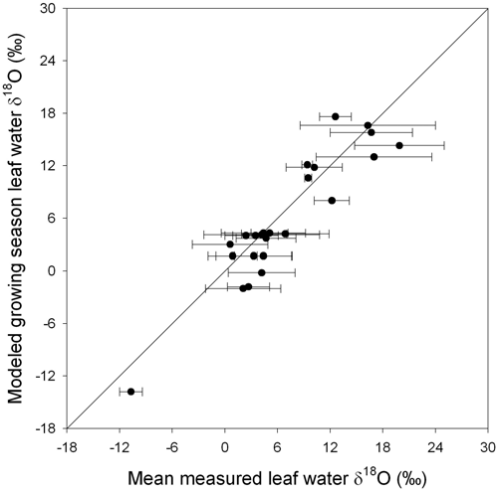
Model comparison with data at known locations. Each point is a mean of reported values (X-axis; error bars are ±1 s.d. and incorporates diurnal and seasonal variability) versus a growing season average evaporative site leaf water prediction derived from the monthly isoscapes (Y-axis). The diagonal line is the 1∶1 line. Growing seasons were defined as May-July for the northern hemisphere (July–August for northern Canada data point), all months for the tropics, and October–December for the southern hemisphere. There was a significant correlation (R = 0.93) between model predictions and mean leaf water. References for the data sources are provided in Appendix S1.

The results of the model comparison to 25 mean values derived from published or unpublished data are shown in Figure 3 (see Appendix S1 for data sources). Clearly for any given site there is a wide range of observed values. However, the mean of these observations at each site fall close to the expected grid-cell growing season averages.

## Discussion

Using spatially continuous data layers and relatively simple models of leaf water isotopic enrichment in a GIS framework, we produced global, spatially continuous leaf water isotope landscapes (isoscapes). These isoscapes represent expected long-term average leaf water isotopic composition. The comparison with zonal averages reported for previous simulation modeling efforts showed that the latitudinal trends in the isoscapes produced here were similar to those observed for the simulation model runs. There were, however, significant differences between all models. As noted previously, these differences highlight the ongoing uncertainties associated with model structures as well as significant uncertainties associated with plant source water and atmospheric vapor isotopic composition. We agree with previous authors that this is a critical area for continued research and data collection. The good agreement between modeled grid-cell growing season averages produced here and the means of specific point measurements strongly suggests that the isoscapes produced here and driven by large-scale continuous maps of climate and water isotope ratios are capturing a significant amount of the existing spatial variability in leaf water δ^18^O.

It is important to place the leaf water δ^18^O results here in the context of previous efforts to model it and the importance of spatial variation in leaf water δ^18^O to several fields of inquiry. Understanding the continental and global spatial patterns of leaf water δ^18^O is critical to accurate interpretation of the isotopic signals in atmospheric gases such as O_2_ and CO_2_ [6], [7], [63], [64], [65]. It is also central to interpreting plant climate proxies [66], [67], and to improving the accuracy of models that use stable isotopes to understand animal diet and migration patterns [1]. Although this is the case, leaf water isotopic enrichment remains one of the more poorly constrained components of global models [6], [20]. Recognizing this inherent uncertainty, we believe that our model predictions for productivity weighted global average leaf water δ^18^O compared well with the range of predictions found in the literature [6], [7], 21. Our predicted global mean leaf water δ^18^O at the sites of enrichment was 6.5‰. This value is 2.1‰ greater than the global mean predicted by Farquhar et al. [7], for the sites of enrichment of 4.4‰, but in agreement with the global average leaf water δ^18^O means of 6.1–6.8‰ (also flux weighted) predicted to be necessary to explain the Dole effect [6]. Values for global mean leaf water δ^18^O as high as 8.7‰ have been reported [21]. Keeling [21] argued that the discrepancy between the leaf water δ^18^O predicted by Farquhar et al. [7] and that required to balance the O_2_ models pointed to a need to create mutually consistent models of both, and emphasized the uncertainty associated with this component of the models. Although our efforts do not resolve these discrepancies, the model comparisons here clearly point to a need to better constrain the spatial and temporal variability of the atmospheric vapor and soil moisture isotopic composition, in particular. As these are better constrained, more comprehensive model comparisons can be made, as well as more intensive comparisons, perhaps at a regional level between point leaf water measurements and model outputs.

In addition to the global averages, it is interesting to compare the latitudinal variation in (un-weighted) leaf water δ^18^O predicted here and those predicted from the (also un-weighted) leaf water isotope fields generated by the ECHAM and GISS models. The latitudinal component of the global spatial variation in the δ^18^O of atmospheric CO_2_ has been of interest for decades, and remains an important component of our attempts to utilize this isotopic signal to understand the coupled carbon and water cycles on Earth [65], [68], [69], [70]. The general latitudinal patterns predicted here were generally consistent with those predicted by Hoffmann et al. [6], but differed sometimes substantially in the latitudinal means predicted. In addition, certain spatial patterns were consistent across all models. For example, all models predict high leaf water δ^18^O values over the Sahara desert and into Saudia Arabia due to the relatively high source water δ^18^O values and high vapor pressure deficit. However, other spatial patterns are not consistent across models suggesting that the GCM models made different climate predictions across spatial domains than the long-term averages used here. Again, improved data density for such important components as vapor and soil water isotopic composition and canopy versus large scale climatology are needed before many of these differences can be resolved.

Some discussion of the potential applications of these now easily-accessible model products and modeling approaches is warranted. Plant organic compounds that are used as climate proxies are also linked to leaf water δ^2^H and δ^18^O through the effects of leaf water on the isotopic composition of the products of photosynthesis [61], [67], [71], [72]. In addition to the large body of work on cellulose δ^2^H and δ^18^O [66], [73], [74], several authors have argued that leaf water ^2^H enrichment is evident in leaf wax δ^2^H [67], [71], [75], suggesting that the combined signal of source water and transpiration may be retained in sediments, and that this may be used to reconstruct past hydrological dynamics. Combined models that explicitly incorporate leaf water isotopic composition in modeling these plant-derived proxies would significantly improve our ability to interpret them. To the extent that leaf water and the products of photosynthesis impart variability in the isotope ratios of animal food sources, these isoscapes can also be useful in the interpretation of animal tissue isotopic signals [76], [77]. Clearly, in addition to the strong spatial patterns observed in these isoscapes, there are large differences in the isotope ratios of the different potential sources of water (e.g., surface water versus leaf water), as well as in the food. These results argue for detailed calibration of models designed to predict animal tissue isotopic composition [78], [79], [80], especially including an understanding of the fractions of H derived from all potential major sources. In addition to their application to animal ecology, there is clear application to forensic reconstructions, especially with respect to identifying the source regions of plant-derived materials [81], [82], [83], [84], [85].

We believe that these results support further exploration of the GIS approach, especially in the context of parallel development and rapid expansion of geo-referenced datasets [86], [87]. A significant advantage of modeling in the GIS environment is that the modeling products can be seamlessly imported and used in additional modeling efforts [e.g., 88], including those that integrate ground-based and remotely sensed data [e.g., 89]. In addition, the models and model products can be easily shared over computer networks. Future work will explore several of the areas of uncertainty discussed, especially with respect to model structure and areas where significant advancements are necessary in the availability of data.

## Supporting Information

Appendix S1 This appendix lists all literature citations used for data comparisons to the model output.
(0.03 MB DOC)
