# Supplementary material for: A Simplified GIS Approach to Modeling Global Leaf Water Isoscapes
Source: PLoS One. 2008 Jun 18;3(6):e2447. doi: 10.1371/journal.pone.0002447 (PMC2413011; doi:10.1371/journal.pone.0002447)
Supplement: Appendix S1 — This appendix lists all literature citations used for data comparisons to the model output. (0.03 MB DOC) [file pone.0002447.s001.doc]

Appendix S1. Sources of δ^18^O data shown in Figure 3.

Barbour MM, Walcroft AS, Farquhar GD (2002) Seasonal variation in δ^13^C and δ^18^O of cellulose from growth rings of *Pinus radiata*. Plant, Cell & Environment 25:1483-1499.

Cernusak LA, Farquhar GD, Pate JS (2005) Environmental and physiological controls over oxygen and carbon isotope composition of Tasmanian blue gum, *Eucalyptus globulus*. Tree Physiology 25:129-146.

Cernusak LA, Pate JS, Farquhar GD (2004) Oxygen and carbon isotope composition of parasitic plants and their hosts in southwestern Australia. Oecologia 139:199-213

Ometto JPH, Flanagan LB, Martinelli LA, Ehleringer JR (2005) Oxygen isotope ratios of waters and respired CO_2_ in Amazonian forest and pasture ecosystems. Ecological Applications 15:58-70.

Muttiah RS, White JD, Duke JR, Allen PM (2005) Estimation of source water to cedar elm in a central Texas riparian ecosystem. Hydrological Processes 19:475-491.

Lai CT, unpublished results from Harvard Forest (42º 32’ N, 72º 11’ W) and Howland Forest (45º 15’ N, 68º 45’ W).

Lai CT, Ehleringer JR, Bond BJ, U KTP (2006) Contributions of evaporation, isotopic non-steady state transpiration and atmospheric mixing on the delta O-18 of water vapour in Pacific Northwest coniferous forests. Plant Cell & Environment 29:77-94.

Flanagan LB, Brooks JR, Varney GT, Ehleringer JR (1997) Discrimination against C-^18^O-^16^O during photosynthesis and the oxygen isotope ratio of respired CO_2_ in boreal forest ecosystems. Global Biogeochemical Cycles 11:83-98.

Pendall E, Williams DG, Leavitt SW (2005) Comparison of measured and modeled variations in pinon pine leaf water isotopic enrichment across a summer moisture gradient. Oecologia 145:605-618

Seibt U, Wingate L, Berry JA, Lloyd J (2006) Non-steady state effects in diurnal O-18 discrimination by *Picea sitchensis* branches in the field. Plant Cell & Environment 29:928-939.

Welker JM, Rayback S, Henry GHR (2005) Arctic and North Atlantic Oscillation phase changes are recorded in the isotopes (delta O-18 and delta C-13) of *Cassiope tetragona* plants. Global Change Biology 11:997-1002.
